# Subclinical Atherosclerosis Progression in Low-Risk, Middle-Aged Adults: Carotid Leads Femoral in IMT Increase but Not in Plaque Formation

**DOI:** 10.3390/jcdd11090271

**Published:** 2024-09-02

**Authors:** Eva Szabóová, Alexandra Lisovszki, Alojz Rajnič, Peter Kolarčik, Peter Szabó, Tomáš Molnár, Lucia Dekanová

**Affiliations:** 1 Department of Angiology, Faculty of Medicine, East Slovak Institute of Cardiovascular Diseases, Pavol Jozef Šafárik University, 040 01 Košice, Slovakia; 2 4th Department of Internal Medicine, Faculty of Medicine, Louis Pasteur University Hospital, Pavol Jozef Šafárik University, 040 01 Košice, Slovakia; 3 Department of Health Psychology and Research Methodology, Faculty of Medicine, Pavol Jozef Šafárik University, 040 01 Košice, Slovakia; 4 Faculty of Aeronautics, Technical University of Košice, 040 01 Košice, Slovakia; 5 Department of Cardiac Surgery, Faculty of Medicine, East Slovak Institute of Cardiovascular Diseases, Pavol Jozef Šafárik University, 040 01 Košice, Slovakia; tmolnar@vusch.sk

**Keywords:** atherosclerosis, carotid artery plaque, carotid intima-media thickness, femoral artery plaque, femoral intima-media thickness, short-term progression of atherosclerosis, ultrasound, vascular risk

## Abstract

This study investigated subclinical atherosclerosis progression in low-risk, middle-aged adults (N = 141; a mean age of 49.6 ± 4.7 years) using a 5-year ultrasound follow-up. We compared the involvement of the carotid and femoral arteries. Methods: Clinical data, risk factors, carotid/femoral intima-media thickness (IMT), and plaque presence were analyzed. Results: Cardiovascular risk factors and scores increased significantly at follow-up. Both carotid and femoral mean IMT increased (*p* < 0.001). While plaque prevalence rose and was similar in both arteries (carotid: 4.8% to 17.9%, femoral: 3.6% to 17.7%, *p* < 0.001 for both), the progression of plaque burden was greater in femorals. Notably, the carotid mean IMT demonstrated a faster yearly progression rate compared to the mean femoral IMT. The prevalence of pathological nomogram-based mean IMT right or left was higher in the carotids (52.9% to 78.8%, *p* < 0.001) compared to femorals (23.2% to 44.7%, *p* < 0.001), with a significant increase at the end of follow-up in both territories. Conclusions: This study demonstrates significant subclinical atherosclerosis progression in low-risk, middle-aged adults over 5 years. Carotid arteries showed a faster progression rate of mean IMT and a higher prevalence of pathological nomogram-based mean IMT compared to the femoral arteries. However, plaque burden was similar in both territories, with greater progression in femorals. Identifying carotid and femoral atherosclerosis burden may be a valuable tool for risk stratification in this population.

## 1. Introduction

Atherosclerosis (ATS) burden is a significant risk for new cardiovascular (CV) events and is related to poor outcomes after CV events. A large proportion of the asymptomatic population stratified by various validated multivariable risk prediction tools is at low-to-moderate CV disease (CVD) risk with missed opportunities for early detection and appropriate management of underlying ATS [1]. The identification of subclinical ATS is an important step in the management of patients in primary CVD prevention. There are several methods to evaluate the presence and progression of subclinical ATS [2,3]. Coronary artery calcification (CAC), carotid intima-media thickness (CIMT), carotid plaque, and ankle-brachial index (ABI) were proposed as valuable markers of subclinical ATS and predictors of CV events, however, with not equal risk redefinition [4]. There is no general consensus whether IMT is a marker of subclinical ATS; rather, it represents arteriopathy [5]. According to the current European guidelines, CAC scoring or, as an alternative when CAC scoring is not feasible, plaque detection by carotid ultrasound (USG) may be considered to improve risk classification around treatment decision thresholds with a IIb B level of evidence [6]. Availability, cost effectiveness, and radiation are the main limitations of using CAC as a screening method with locoregional consideration [6]. Arterial stiffness parameters are non-invasive functional markers of hypertension-mediated organ damage—CV risk modifiers. However, measurement difficulties and publication bias argue against their widespread use [6]. Since ATS is a global disease, the study of ATS requires a multimodal and multiterritorial approach. Several studies support the value of measuring subclinical ATS in multiple arterial territories for a more accurate CV risk stratification [7,8]. Subclinical ATS is highly prevalent in the middle-aged asymptomatic population [9,10,11]. In addition, clinical data documented extensive ATS in a substantial number of low-risk individuals [9]. Except for the preferentially screened carotid and coronary areas, the iliofemoral arteries and abdominal aorta are also frequently affected. Results from the Progression of Early Subclinical Atherosclerosis Study (PESA) documented an even higher prevalence of ATS plaque in the iliofemoral arteries compared with the carotid, abdominal, and coronary arteries [9]. The identification of global atherosclerotic burden is a useful tool to identify patients at high CVD risk. There are a limited number of studies comparing the presence and progression of subclinical ATS in different arterial regions [12]. Ultrasound-based techniques are non-invasive, accessible, quick, easily trained, low-cost, and radiation-free [13], so they are suitable for population screening. We aimed to study the short-time progression rate of carotid and femoral subclinical atherosclerosis in middle-aged, apparently healthy individuals to evaluate ultrasound-based techniques’ potential use in primary prevention.

## 2. Patients and Methods

The present study is an observational, prospective, real-life study of a target, addressed population of 400–450 apparently healthy subjects. The study subjects were 141 participants of Caucasian origin without established CVD, 56.7% women and 43.3% men, aged 49.6 ± 4.7 years, who underwent baseline and 5-year follow-up (4.67 ± 0.95 years) visits between February 2010 and October 2017. The study design has been reported elsewhere [11]. Briefly, non-diabetic males or females 35–55 years of age inhabiting the East Slovak Region, with obtained written informed consent, were included. Subjects with established CVD; European Systematic Coronary Risk Evaluation (SCORE) risk ≥ 5%; chronic kidney, respiratory, or hepatic disorders; neoplasia; severe obesity (body mass index (BMI) > 35 kg/m^2^); alcoholism; non-compliance; pregnancy; as well as acute inflammatory disorders were excluded. Out of the target population, only 256 persons met the inclusion criteria. We excluded 69 subjects, mainly because of high SCORE risk, ECG pathology, newly diagnosed DM, pathological urinary findings, and renal abnormalities confirmed at baseline assessment. Finally, 187 individuals were enrolled in the study, 141 of them (75.4%) finished the follow-up; the others were contacted, but they did not show interest in continuing the study. During the follow-up, we observed one sudden cardiac death (0.53%), one suicidal death, and one nonfatal CV event (unstable angina pectoris). This study was conducted in accordance with the Declaration of Helsinki, and the protocol was approved by the Ethical Committee of the L. Pasteur University Hospital in Košice (approval number 2020/EK/02018).

## 3. Data Collections and Statistics

### 3.1. Data Collection

Participants were examined in the Outpatient Department of the 4th Clinic of Internal Medicine at L. Pasteur University Hospital in Košice in the morning under basal conditions. The examination itself consisted of the blood and urine collection for biochemical analysis, the detection of morphological markers of subclinical vascular damage; interviews for medical history with the focus on classical risk factors for ATS and current medications; measurements of body size, waist circumference, and office blood pressure; the determination of 10-year fatal and total CV risk (European SCORE), and resting 12-lead electrocardiogram recording. Blood and urine samples were analyzed in the relevant subdivisions of the department of laboratory medicine at the same hospital. Metabolic parameters used in our work (fasting glucose, glycated hemoglobin (HbA1c), uric acid, serum total cholesterol (T-C), high-density lipoprotein cholesterol (HDL-C), low-density lipoprotein cholesterol (LDL-C), triglycerides (TAG), serum creatinine) were directly determined by standard laboratory tests; estimated glomerular filtration rates (eGFRs) were calculated according to the Modification of Diet in Renal Disease (MDRD) formula [14]. The following values were considered pathological: creatinine > 90 µmol/L, eGFR < 1.5 mL/s/m^2^, and uric acid > 357/428 µmol/L (males/females). Non-modifiable risk factors for ATS as well as arterial hypertension (AH), dyslipoproteinemia (DLP), obesity/central obesity, diabetes mellitus (DM), impaired fasting glucose, and metabolic syndrome (MetS) were defined according to current recommendations [15,16]. Smoking status was characterized as current smoking ≥ 1 cigarette/day. To estimate a person’s 10-year risk of CV death, we used the SCORE chart for high-risk countries (low risk < 1%/moderate risk ≥ 1% and <5%); the total 10-year CV event risk was calculated by multiplying fatal risk (3× for men and 4× for women) [15]. The targeted dietary and pharmacological management of AH and DLP was satisfactory at the time of patient enrollment into the study, and with a few exceptions, the therapeutic goals were achieved throughout the entire study. No polypharmacotherapy was observed; the subjects were treated mainly with one prescribed drug and occasionally in combination with a single pill. The most commonly prescribed antihypertensive drugs were ACE inhibitors/sartans, followed by calcium channel blockers and thiazide/thiazide-like diuretics used mostly in combination, as well as beta-blockers in specific indications. DLP was treated with statins (titrated dose) or, if indicated, by ezetimibe. Based on personalized CV risk assessment, preventive measures (lifestyle modifications and/or pharmacological treatment) were recommended for each subject, to which they agreed. Adherence to instructions was regularly checked by family doctors and study investigators.

### 3.2. Morphological Markers of Subclinical Arteriopathy

#### 3.2.1. Carotid IMT and Plaque Assessment

Ultrasonography was performed by one experienced sonographer with acceptable intraobserver variability of measurements, blinded to each subject’s health status and risk factors. Details of the USG methodology and quality control have been reported previously [11,17]. CIMT and carotid plaque were defined according to the Mannheim consensus [18,19]. Bilateral carotid arteries were scanned using high-resolution B-mode USG (Philips HD 15) with the 7.5 MHz probe in real-time at 5× magnification. IMT was defined as the distance from the leading edge of the lumen–intima interface to the leading edge of the media–adventitia interface and was measured on a distinct plaque-free segment of the common carotid artery (CCA) far wall, 1 cm from the flow divider, in the end-diastole, at its presumed maximum thickness. Examinations were made automatically. ATS plaque was defined as an endoluminal protrusion of at least 1.5 mm or a >50% focal thickening of the IMT relative to the adjacent wall segment. Plaque presence on both transverse and longitudinal planes was recorded in the CCA, bulb, and internal (ICA) and external (ECA) carotid arteries. Generally, the carotid plaques were stable and isoechogenic and had smooth surfaces and normal peak systolic velocities (PSVs) at baseline and during follow-up visits. The following CCA parameters were evaluated in our work: the mean value of CIMT separately on the right and left (CIMTdx, sin); the maximum value of CIMT right or left (CIMTmax); CIMT > 0.9 mm right or left (CIMTbilat of >0.9 is considered abnormal, although the upper limit of normality varies with age [20,21]); pathological nomogram-based mean right or left CIMT by age and sex (asCIMTbilat), i.e., on the left side in males/females aged 31–40 years, asCIMTbilat > 0.57/0.51 mm, 41–50 years, asCIMTbilat > 0.61/0.57 mm, and over 50 years, asCIMTbilat > 0.70/0.64 mm and on the right side, in males/females aged 31–40 years, asCIMTbilat > 0.5/0.49 mm, 41–50 years, asCIMTbilat > 0.57/0.53 mm, and over 50 years, asCIMTbilat > 0.62/0.59 mm [22,23]; CCA-IMT progression (mm/year); and the presence of carotid plaque.

#### 3.2.2. Femoral IMT and Plaque Assessment

The literature diverges on the issue of the reference measurement site and methodology of IMT (especially in areas other than the carotid); even the pathological values of IMT in the carotid or femoral area are not uniform. Considering the need to use the same methodology, we proceeded with the assessment of subclinical arteriopathy of the femoral artery as in the carotid area. The definition of IMT and plaque was identical for both arterial territories. Bilateral common femoral arteries (CFAs) were scanned, and femoral IMT (FIMT) was obtained 1–2 cm proximal from the bifurcation on the far wall of the CFA [24]. For the plaque presence, the CFAs, the superficial and profundal femoral arteries were examined for a length of 3 cm (1.5 cm proximally and distally to the flow divider) [7]. The following CFA parameters were evaluated in our work: the mean value of FIMT separately on the right and left (FIMTdx, sin); the maximum value of FIMT right or left (FIMTmax); FIMT > 0.9 mm right or left (FIMTbilat > 0.9) [25]; FIMT > 1.1 mm right or left (FIMTbilat > 1.1) [24]; nomogram-based pathological mean right or left FIMT by age and sex (asFIMTbilat), i.e., in white males/females aged 24–43 years, asFIMTbilat > 0.75/0.64 mm [26]; CFA-IMT progression (mm/year); and presence of femoral plaque.

### 3.3. Statistical Analysis

Patient’s data were summarized at baseline and at the end of follow-up and analyzed by means of descriptive statistical methods. Continuous variables are shown in the tables in the form of arithmetic mean and standard deviation (SD), and the categorical variables are shown as absolute numbers and their relative representations (%) in the sample. An analysis of differences in the continuous clinical parameters investigated, including markers of subclinical vascular damage between patients at baseline and at follow-up visits, was carried out using a paired samples *t*-test. A McNemar’s test was used to compare the frequencies of categorical variables in time between paired samples. During the follow-up, a progression rate of mean CIMT and FIMT was also calculated. To describe the individual changes of assessed parameters during the follow-up, we calculated the percentage change relative to the baseline parameter. A value of *p* < 0.05 was considered statistically significant. The analyses were performed using the IBM SPSS 23.0 statistical software package (IBM Corp., Armonk, NY, USA).

## 4. Results

### 4.1. Characteristics of the Study Group

Out of the study sample of 187 initially enrolled individuals, 141 persons were checked after a follow-up. Demographic, clinical, and laboratory data at baseline and after follow-up are shown in Table 1.

### 4.2. Risk Profile and Subclinical Carotid or Femoral Arteriopathy Burden at Baseline and at Follow-Up

Changes in the person’s risk profile, including analyzed structural markers of subclinical arteriopathy after 5 years, are listed in Table 2. After follow-up, we documented a significantly higher prevalence of modifiable risk factors: DLP, central obesity, AH, as well as a corresponding increase in SCORE risk (1.2 ± 1.61; *p* < 0.001) and the number of risk factors (3.72 ± 5.82; *p* < 0.05). The mean values of CIMT right and left (0.62 ± 0.10 mm; *p* < 0.001 for both) were significantly increased but remained under the “abnormal level of 0.9 mm—previously identified as hypertension-mediated organ damage” [20,21] at follow-up. The increases in mean (0.07–0.08 ± 0.12 mm) and maximum (0.07 ± 0.13 mm) values of CIMT were significant. The mean and maximum values of IMT at baseline and at follow-up were almost identical at carotid and femoral sites (Table 2). The mean right and left CCA-IMT change/year was the same: 0.017 ± (0.027–0.029) mm. The FIMT progression was slower in comparison with CIMT, with the lowest rate for FIMTdx (right—0.0085 ± 0.035 mm/year; left—0.012 ± 0.044 mm/year). The occurrence of CIMT > 0.9 mm was rare (2.1%) and did not change significantly during the follow-up. In comparison with the carotid region, the FIMT > 0.9 mm was more frequent at the first and last visits; on the other hand, the presence of the other femoral IMT cut-off value, FIMT > 1.1 mm, was similar to the carotid region, i.e., rare. However, the prevalence of asCIMTbilat was higher (78.8%), with a greater increase (+25.9%) at the end of follow-up, in comparison with the occurrence and increase rate of asFIMTbilat (44.7% and +21.5%, respectively). Similar significant increases in the rates of carotid and femoral plaque burden were also observed (from 4.8% to 17.9% and 3.6% to 17.7%, respectively; *p* < 0.001 for both), but with higher progression in femorals (13.1% vs. 14.1%). Initially, in 8.4% of the subjects, we found carotid or femoral plaque; at the end of the follow-up, it was 26.9% (in 12 subjects, plaques were present in both the carotid and femoral regions). If only the carotid area was examined, the ATS plaque on the femoral artery would be missed in approximately 9% of patients at the end of follow-up. It is interesting to observe the rate of exact change in the risk profile of our group over the course of 5 years (Table 2). The mean and maximal values of CIMT and FIMT were moderately changed. On the other hand, an important change occurred in SCORE risk (103%), in the proportion of pathological nomogram-based mean CIMT and FMT (49% and 93%, respectively), but the biggest changes were recorded in the occurrence of ATS plaques—270–390%.

## 5. Discussion

The examination of different arterial segments may complement each other in the evaluation of the presence and extent of ATS and in the modification of CV risk [27]. There is a limited number of studies comparing the progression of subclinical ATS in different regions [12] with the impact on the timing of population screening.

In our 5-year prospective study, we found similarities and differences in the short-term progression of subclinical carotid and femoral arteriopathy. The increases in mean and maximum values of CIMT and FIMT were significant, with low and almost identical CIMT and FIMT values. The yearly progression rate of IMT was slower in the femoral region in comparison with the carotids. IMT > 0.9 mm (previously identified as hypertension-mediated organ damage) was five times more frequent in the femoral region in comparison with the carotids. On the other hand, the occurrence of FIMT > 1.1 mm (predictive value of CIMT > 0.9 mm) [24] was as low as CIMT > 0.9 mm. The presence of pathological nomogram-based mean CIMT and FIMT was surprisingly high (mainly carotid), and compared to the beginning of the study, the prevalence was significantly higher by 25.9%. Similarly, a relatively high and similar prevalence of carotid (17.9%) and femoral (17.7%) plaque burden was documented at the end of follow-up, with a more pronounced progression during the follow-up in femoral region, where a substantial change in the occurrence of femoral plaque was observed compared to the baseline (an almost 400% change). The disproportionality of changes in the SCORE value and the occurrence of ATS plaques (mainly in the femoral area) confirms the importance of personalized screening.

### 5.1. Risk Profile

The risk profile of our study group is comparable with that in the literature [10,28] and was commented in our previous study [11]. Obesity and DLP were increased because we followed central obesity and tighter cut-offs for DLP. In the large ongoing PESA study with enrollment of participants without CVD, with no exclusion of diabetics, the study group had a better risk profile in terms of DLP (40.9%) and obesity (13.3%), but the proportion of lipid-lowering therapy was similar (6.6%) [29]. Changes in creatine values were evaluated as the physiological variations; the patients did not show signs of renal disease throughout the study. The estimated GFR value uses the same analytical (interference) and biological limits as the determination of serum creatinine. According to the calculation of eGFR, the lower creatinine level is associated with a falsely higher GF level. In addition, at the end of the study, we had the same subjects with hyperfiltrations, which could modify the eGFR value.

### 5.2. CIMT and FIMT Progression

Increased CIMT represents subclinical vascular disease and CVD risk marker [30,31], may be related to intimal and/or medial hypertrophy, and may be an adaptive response to changes. Increased CIMT is related to (not clearly synonymous with) subclinical ATS because of similar alterations in the progression of both processes [30]. This is why there is a shift in this context in the terminology from subclinical ATS to arteriopathy in a couple of recent articles [5]. The initiation, progression, and expression of ATS lesions are mainly artery-related [32]. Shared common risk factors have different impact sin different arterial territories [33]. Autopsy studies revealed that, in different vascular segments, there is no uniform involvement of ATS [34]. ATS plaques in different segments of the arterial tree have similar cell types, but their relative amounts of connective tissue and lipids can vary considerably [35]. Twin studies also reported a heritable component on carotid and femoral IMT [36]. Like carotid, femoral artery wall morphology is correlated with subclinical ATS, is associated with CAC score (CACS) [8], and is an independent predictor of future CV events [37,38,39]. Some studies have reported that ATS changes are more advanced in the femoral than carotid artery [40]; other ones revealed that the IMT of the femoral artery is a better indicator of the extent and severity of coronary artery ATS than in carotid arteries [41]. Based on the different progression of ATS plaques and IMT in the carotid and femoral areas, it is possible to assume, rather, hypertension (central pressure) induced increase in IMT in the carotid region. The examination of various arterial segments may complement each other in the evaluation of the extent of ATS [27]. The majority of studies have assessed only common carotid artery IMT. The USG of femoral arteries for CV risk modification has not become a part of the routine. Moreover, comparative data from the presence and dynamics of vascular target organ damage phenotypes in carotid and femoral arterial segments are scarce [40].

A systematic review reported the mean CIMT between 0.62 and 1.07 mm and CIMTmax between 0.78 and 1.8 mm in low-to-intermediate risk individuals aged 60 ± 7.6 years [42]. In the PESA study, with a comparable mean age of the study population, similar to our results [11], the mean CIMT value was 0.59 mm [9,29]. The varying progression rate of the mean CCA-IMT published in different population-based studies ranged between 0.0038–0.060 mm/year [43,44]; other studies detected comparable progression rate to ours [45,46]. A mildly higher rate of CCA-IMT (0.025 mm/year) was observed in the large Atherosclerosis Risk in Communities (ARIC) study [47]; a lower progression rate of the CCA-IMT was documented in the Carotid Atherosclerosis Progression Study (CAPS) (0.001 mm/year) [28].

For the CVD risk assessment, instead of normative values (i.e., pathological IMT > 0.9 mm, reflecting primarily ATS at the carotid bifurcation and hypertension-mediated hypertrophy at the level of CCA), carotid USG imaging and measurements should follow protocols with CIMT values in percentiles by age, sex, race/ethnicity, and mostly left/right [22,23,48]. In comparison with previous data [10], the occurrence of CIMT > 0.9 mm was rare in our study and not significantly changed after the 5-year follow-up [11]. Similar to our results, CIMT > 0.9 mm was detected in 1% of participants in the PESA study [9,29]. In contrast, there was a 36.7% incidence of CIMT > 0.9 mm reported by Mitu et al. among apparently healthy individuals, classified mainly in high-risk SCORE [10].

Similar to our results [11], the 75th percentile of the CCA-IMT distribution was established at 0.58 and 0.59 mm in healthy females and males, respectively, without CV risk factors, over 40 years of age [49]. The prevalence of CIMT > 75th percentile for the patient’s age, sex, and race/ethnicity was approximately 12% across the Framingham Heart Study, but for the intermediate Framingham risk score (FRS), 22–58% of patients had increased CIMT [50]. However, no data are available on the progression rate of pathological nomogram-based mean CIMT in the literature.

Very similar mean FIMT values to ours were found by Deparion et al. [51] in healthy subjects aged 20–60 years without CV risk factors: 0.543 ± 0.063 mm and 0.562 ± 0.074 mm for women and men, respectively. The estimated increase per year was less than in our study, (0.0031 mm for men and 0.0012 mm for women), probably because they screened subjects without CV risk factors. In our study, the presence of risk factors was not an exclusion criterion. In a size and risk profile similar study to ours, regardless of sex, the mean FIMT was 0.80 ± 0.2 mm, higher than in our study [40]. A bit higher mean FIMT (0.64 mm females /0.75 mm males) was measured in healthy participants of the Bogalusa Heart Study (aged 24–43 years) [26]. The population-based, low-risk French AXA Study (Sex and Topographic Differences in Associations Between Large-artery Wall Thickness and Coronary Risk Profile in a French Working Cohort) in employees of the insurance company AXA France, aged 17–65 years, with no exclusion of CVD and CV risk factors, the documented mean FIMT was 0.43 ± 0.06 mm for women and 0.50 ± 0.11 mm for men (thinner than FIMT in our study), with progression rates of 0.003 and 0.005 mm/year for women and men, respectively [52].

The pathological nomogram-based mean femoral IMT occurrence, FIMT > 0.9 mm, has received less attention to date in the literature. Langlois et al. found the maximal FIMT to be 0.59 (0.51–0.70) mm in females and 0.71 (0.60–0.87) mm in males [25], which is similar to our results. In the same cohort [25] with no exclusion of DM, 26.3% of subjects had FIMT > 0.9 mm, more than in our study.

In a population of 156 apparently healthy normotensive Caucasian volunteers between 18 and 65 years, Rietzschel et al. revealed similar results to us: identical right common femoral and carotid mean IMT (0.52 mm) [53]. In one above-mentioned study [40], the mean and maximal femoral IMTs were greater than the mean and maximal carotid IMT. In accordance with us, the CIMT was greater in other studies [25,26]; also, the progression rate was higher for CIMT than for FIMT in the AXA study and in the study conducted by Markus [52,54].

### 5.3. Carotid and Femoral Plaque Progression

Carotid IMT and plaque are markers for measuring ATS burden that are strongly associated with vascular risk factors and the incidence of CV events [31]. ATS progression predicts CV events [55]. The occurrence of carotid plaques is variable in the general population and may be explained by age, CV risk factors, and geographical influence [10]. According to a systematic review [42], the occurrence of plaque in asymptomatic, low-to-intermediate-risk cohorts with different age and risk profiles was an average of 35%. Some authors [10], in comparison to our results, reported a higher prevalence of carotid plaque (40%), probably due to the enrollment of older subjects. Studies with asymptomatic, middle-aged individuals documented higher occurrence of carotid plaques (29.3% in subjects with risk SCORE < 5% [10], 31% in the PESA study [29]).

There is a slight difference in the genesis of ATS plaques in CCA and CFA, supported by pathology [35], biochemical studies [25], different distributions of plaques in carotid and femoral sites [35], as well as by significant side differences in the IMT of CFA but not of CCA, underlining a possible role of local geometry in the development of ATS [56]. However, this side difference was not observed by us, not even in the carotid area.

Although ATS is considered a generalized disease process, the extent of ATS and its underlying risk factors differ among arterial sites, confirmed by autopsy studies [34]. It has been shown that ATS lesions are more frequent and advanced in femoral arteries than in carotid arteries, independent of the number of risk factors [57,58]. ATS in femoral arteries occurred earlier than carotid arteries [57], and the femoral artery is more susceptible to the atherogenic influence of risk factors [58].

In our study, the occurrence of carotid plaque was slightly higher than femoral plaque, mainly at baseline; the difference practically disappeared at the end of follow-up because of the higher progression rate in the femoral region. Among PESA participants, plaques were most common in the iliofemorals (44%), followed by the carotids (31%), aorta (25%), and coronary arteries (18% [9,29]). Interestingly, among participants with low Framingham Heart Study (FHS) 10-year risk, subclinical disease at all was detected in 58% (higher than in our study but in multiterritorial locations). Nearly 60% of those with CACS = 0 had plaques at other vascular sites, implying that, in the low-risk sample, the absence of CAC does not necessarily indicate that a participant is disease-free [9]. In the Cafes-Cave 10-year prospective study with 10,000 healthy, low-risk individuals without AH, DM, and DLP, aged 35–65 years, 10.8% of the study population had ATS plaque at either the femoral or carotid level (less than in our study, but their study population was free of the three main modifiable risk factors). Moreover, the authors documented a difference in morphology between carotid and femoral arteries: in 51% of subjects, the carotid was the most advanced artery, and in 52.4%, the right (carotid or femoral) arteries were more advanced than the left [7]. We observed almost the same prevalence on both arterial sites but did not evaluate side differences.

## 6. Limitations

Limitations of our study are a small study group and low response rate (75%). Moreover, the lack of methodological standardization, measurement difficulties, and publication bias make it difficult to compare our results with other studies, mainly carotid and femoral IMT parameters. In addition, there are limited data focusing on the comparison of subclinical ATS/arteriopathy progression incorporating two peripheral arterial sites concurrently in similarly selected subjects and using markers. Because of these limitations, there is a need for cautious interpretation of our results. Additional research in a larger sample of asymptomatic individuals is needed to quantify the impact of imaging in different arterial territories for subclinical vascular damage in CV risk management before applying them in clinical practice.

## 7. Conclusions

In middle-aged, non-diabetic, low-to-moderate CV-risk individuals, during a short follow-up, a relatively high prevalence and a significant progression of subclinical carotid and femoral ATS/arteriopathy were detected by standardized ultrasound techniques, expressed mainly as the presence of plaque and increase in the pathological nomogram-based mean carotid and femoral IMT. Carotid arteries showed a faster progression rate and higher prevalence of pathological nomogram-based mean IMT compared to the femoral arteries. However, plaque burden was similar in both territories, with a higher progression rate in femorals. The high prevalence and varied short-term dynamics of subclinical ATS/arteriopathy in the carotid and femoral regions (between 45 and 50 years of patients’ age), supported by clinical studies confirming the lower effectiveness of treatment in patients with advanced ATS/arteriopathy [59,60], may underline the importance of early screening for subclinical carotid and femoral arteriopathy and optimal timing of personalized CV risk stratification with subsequent optimal management in middle-aged subjects with low-to-moderate calculated CV risk, especially in those over 50 years old with several risk factors.

## Figures and Tables

**Table 1:** Comparison of mean values, standard deviations (SDs), changes (Δ) and % change of continuous anthropometric, clinical and biochemical data at baseline and after follow-up assessed with paired *t*-test.

Parameter	Baseline	Follow-Up	Δ	% Change	*p*
	N = 141 Mean (SD)	N = 141 Mean (SD)	Mean (SD)		
Age (yr)	45.64 (5.02)	49.64 ( 4.67)	4.35 (1.6)	8.76	<0.001
Waist circumference (cm)	87.63 (13.07)	92.33 (12.87)	4 (5.39)	5.36	<0.001
BMI (kg/m^2^)	25.28 (3.89)	25.67 (4.55)	0.38 (1.48)	1.54	0.003
Total cholesterol (mmol/L)	5.47 (0.93)	6.00 (1.09)	0.48 (0.88)	9.69	<0.001
LDL-C (mmol/L)	3.24 (0.79)	3.91 (0.83)	0.63 (0.75)	20.68	<0.001
HDL-C (mmol/L)	1.5 ( 0.35)	1.47 (0.36)	−0.01 (0.21)	−2.00	NS
Triglycerides (mmol/L)	1.26 (0.74)	1.47 (0.86)	0.15 (0.56)	16.67	0.002
Plasma glucose (mmol/L)	5.01 (0.47)	5.13 (0.49)	0.11 (0.4)	2.40	0.001
HbA1c (IFCC) (mmol/mol)	34.4 (3.6)	32.4 (3.5)	−1.9 (3.4)	−5.81	<0.001
Uric acid (µmol/L)	297.27 (80.09)	312.16 (81.9)	13.97 (45.31)	5. 01	0.001
Creatinine (µmol/L)	86.45 (10.64)	71.36 (11.91)	−16.36 (5.63)	−17.46	<0.001
eGFR (mL/min/1.73 m^2^)	70.2 (7.8)	96.6 (11.4)	26.4 (9.0)	37.61	<0.001

Remarks: BMI, body mass index; LDL-C, low-density lipoprotein cholesterol; HDL-C, high-density lipoprotein cholesterol; HbA1c, glycated hemoglobin; IFCC, International Federation of Clinical Chemistry and Laboratory Medicine; eGFR, estimated glomerular filtration rate; NS, statistically nonsignificant difference; N, number; SD, standard deviation; Δ, change; *p*, statistical significance; yr, years.

**Table 2:** Comparison of prevalence and mean values of cardiovascular risk factors and morphological markers of subclinical carotid/femoral arteriopathy at baseline and after follow-up assessed with McNemar’s or paired *t*-test. The comparison of changes and % change in parameters is at baseline and after follow-up.

Parameter	Baseline	Follow-Up	Δ	% Change	*p*
	N = 187 **/141 * Mean (SD)	N = 141 **/141 * Mean (SD)	Mean (SD)		
Risk age (N/%)	41/21.9	65/46.1	24/24.2	110.5	NS **
Sex (male) (N/%)	75/40.1	61/43.3	−14/3.2	7.9	NS **
Positive family history (N/%)	33/17.8	31/22.1	−2/4.3	24.1	NS **
DLP (N/%)	132/71	126/89.4	−6/18.4	25.9	<0.001 **
AH (N/%)	48/25.8	54/38.6	6/12.8	49.6	<0.001 **
Duration of AH (years)	0.78 (2.12)	2.1 (4.57)	1.32/(2.45)	169.23	<0.001 *
Smoking (N/%)	38/20.3	28/19.9	−10/−0.4	−1.9	NS **
MetS (N/%)	31/16.8	40/28.4	9/11.6	69.0	NS **
Central obesity (N/%)	105/57.4	103/74.6	−2/17.2	29.9	<0.001 **
SCORE fatal	0.57 (0.93)	1.16 (1.56)	0.59/(0.63)	103.51	<0.001 *
SCORE total	1.81 (2.70)	3.71 (4.72)	1.9/(2.02)	104.97	<0.001 *
Number of risk factors	2.61 (1.63)	3.78 (6.06)	1.17/(4.43)	44.83	<0.027 *
Treatment of DLP (N/%)	12/6.4	12/8.5	0/2.1	32.8	NS **
CIMT sin (mm)	0.54 (0.09)	0.62 (0.10)	0.08 /(0.11)	14.81	<0.001 *
CIMT dx (mm)	0.54 (0.09)	0.62 (0.10)	0.08 /(0.12)	14.81	<0.001 *
CIMT max (mm)	0.67 (0.11)	0.74 (0.11)	0.07 /(0.12)	10.45	<0.001 *
CIMT bilat > 0.9 mm (N/%)	2/1.1	3/2.1	1/1.0	90.9	NS **
asCIMT bilat (N/%)	99/52.9	111/78.8	12/25.9	48.9	<0.001 **
Carotid plaque (N/%)	9/4.8	25/17.9	16/13.1	272.9	<0.001 **
FIMT sin (mm)	0.56 (0.13)	0.64 (0.14)	0.08/(0.17)	7.14	<0.001 *
FIMT dx (mm)	0.56 (0.14)	0.63 (0.15)	0.07/(0.15)	12.5	<0.001 *
FIMT max (mm)	0.70 (0.15)	0.79 (0.17)	0.09/(0.18)	12.86	<0.001 *
FIMT bilat > 0.9 (N/%)	7/5.1	16 /11.4	9/6.3	123.52	NS **
FIMT bilat > 1.1 (N/%)	3/2.2	4 /2.9	1/0.7	31.8	NS **
asFIMT bilat (N/%)	32 /23.2	63/44.7	31/21.5	92.7	<0.001 **
Femoral plaque (N/%)	5/3.6	25/17.7	20/14.1	391.7	<0.001 **

Remarks: DLP, dyslipoproteinemia; AH, arterial hypertension; MetS, metabolic syndrome; CIMT dx/sin/max, mean right/mean left/maximum common carotid artery intima-media thickness; FIMT dx/sin/max, mean right/mean left/maximum common femoral artery intima-media thickness; CIMTbilat > 0.9 mm, common carotid artery intima-media thickness > 0.9 mm right or left; FIMT bilat > 0.9 mm, common femoral artery intima-media thickness > 0.9 mm right or left; FIMT bilat > 1.1 mm, common femoral artery intima-media thickness > 1.1 mm right or left; asCIMTbilat, nomogram-based pathological mean common carotid artery intimamedia thickness by age and sex on the right or left; asFIMT bilat, nomogram-based pathological mean common femoral artery intima-media thickness by age and sex on the right or left; SD, standard deviation; NS, statistically nonsignificant difference; N, number; *p*, statistical significance; *, paired t-test; **, McNemar’s test; Δ, change. In paired *t*-test, N = 141 at baseline and follow-up; in McNemar’s test, N = 187 at baseline and N = 141 at follow-up. Within statistical test “% change” the calculations in categorical parameters were made with percentages.

## Data Availability

The data presented in this study are available on request from the corresponding author.
